# Vaccination Barriers in Brazil: Exploring Hesitancy, Access, and Missed Opportunities in a Cohort of Children (2017–2018)—National Vaccination Coverage Survey Results (2020–2021)

**DOI:** 10.3390/vaccines13050516

**Published:** 2025-05-13

**Authors:** Letícia Bezerra Faria, Ana Paula França, José Cássio de Moraes, Maria Rita Donalisio

**Affiliations:** 1 Faculty of Medical Sciences of the University of Campinas, Campinas 13083-887, Brazil; leticiabezerrafaria@gmail.com; 2 Faculty of Medical Sciences of the Santa Casa de São Paulo, São Paulo 01224-001, Brazil; ana.franca@fcmsantacasasp.edu.br (A.P.F.); jcassiom@uol.com.br (J.C.d.M.)

**Keywords:** vaccination, vaccination hesitancy, miss opportunity of vaccination, cohort study, children

## Abstract

Background/Objectives: In recent years, Brazil has experienced declining vaccination coverage, raising concerns about vaccine hesitancy and barriers to access. This research analyzes the reasons for non-vaccination among children born in 2017 and 2018 in a metropolitan area of the state of São Paulo in 2020 and 2021. Methods: Data were obtained from a retrospective cohort of children born in 2017 and 2018, living in Campinas, monitored during the first 24 months by vaccination records. A stratified and clustered sample by census sector was performed according to socioeconomic conditions. The reasons for non-vaccination were obtained from interviews with the children’s guardians. Results: A total of 1775 caregivers were interviewed, and 63.1% of children had complete vaccination coverage, with lower socioeconomic groups presenting the highest rates for non-vaccination. The study identified three main groups for non-vaccination: vaccine hesitancy (e.g., fear of side effects, misinformation) in 1.7% of respondents, access difficulties (e.g., service location, financial constraints) in 7.9%, and missed opportunities (e.g., lack of vaccines, administrative barriers) in 16.4%. Conclusions: The findings indicate that the main reported barriers to childhood vaccination are missed opportunities in healthcare services, often due to vaccine shortages or administrative issues, along with social vulnerabilities. Vaccine hesitancy stems from misinformation and fear of side effects. Despite these challenges, families persist in seeking vaccination. However, coverage remains below the national targets, particularly in the second year of life. Targeted public health interventions are urgently needed to improve vaccine confidence, accessibility, and healthcare system efficiency.

## 1. Introduction

Vaccination is a key strategy for preventing and controlling infectious diseases, significantly reducing morbidity and mortality in populations. It is considered one of the most cost-effective health investments. New platforms and production technologies have helped improve vaccines, making them safer [1,2]. Brazil is internationally recognized for its high vaccination coverage and success in controlling vaccine-preventable diseases, led by the National Immunization Program (Programa Nacional de Imunização).

In 1973, the Brazilian National Immunization Program (PNI) was established by the Ministry of Health to develop integrated strategies that expanded immunization coverage across the country, aiming to control vaccine-preventable diseases. Since then, significant progress has been observed in vaccination coverage.

A study that analyzed the average vaccination coverage in Brazil for vaccines recommended within the first year of life between 2013 and 2020 observed that in 2020, the coverage was 75.1%, the lowest rate recorded since 2013 (98.9%). During this period, there were fluctuations in the rates, with the highest increase in coverage occurring between 2017 (85.9%) and 2018 (89.6%), but still below the targets. On the other hand, the most significant drops were recorded between 2015 (95.9%) and 2016 (90.0%), and even more sharply between 2019 (84.4%) and 2020 (75.1%) [3].

However, considering the declining vaccination coverage for most vaccines in recent years, it is necessary to better understand the reasons behind this decrease and the growing vaccine hesitancy observed in Brazil, as in other parts of the world [4,5]. Vaccination coverage surveys have played a strategic role in identifying trends and factors associated with non-vaccination, as well as facilitating the monitoring of vaccination coverage in the country [6,7].

Although we see a reversal of the declining trend and a prioritization of vaccination coverage by the Ministry of Health in the past year, numerous factors remain associated with hesitancy and lack of access to vaccination.

Vaccine hesitancy refers to delays in accepting or refusing vaccination despite their availability in healthcare services. Vaccine hesitancy is complex and context-specific, varying over time, location, and vaccine type. It is influenced by factors such as complacency, convenience, and confidence [8,9]. Socioeconomic factors and difficulties in accessing healthcare may contribute to incomplete vaccination schedules [7,10]. Additionally, missed vaccination opportunities within healthcare services are also a constant challenge for Brazil’s Unified Health System (SUS).

Thus, this research aimed to analyze the reasons for non-vaccination, including vaccine hesitancy, lack of access, and missed opportunities, in the city of Campinas, São Paulo, as well as the factors associated with these barriers in order to contribute to public health measures and overcome these challenges.

## 2. Materials and Methods

This research analyzed data from a retrospective cohort of children born in 2017 and 2018, obtained from the National Vaccination Coverage Survey (INCV), a population-based survey conducted in a metropolitan area of the state of São Paulo in 2020 and 2021, funded by the Brazilian Ministry of Health. The household survey in the urban area involved interviews with caregivers and photographic documentation of vaccination cards to assess the completeness of vaccination schedules recommended by the Ministry of Health and identify the reasons for non-vaccination [6,11].

### 2.1. Study Location

This research was conducted in the city of Campinas (population: 1.2 million) (IBGE, 2023), a regional medical and hospital reference center with a Human Development Index (HDI) of 0.805 (very high). As of December 2020, 196 teams in Campinas were linked to the Family Health Strategy (ESF), covering 56.2% of the population and providing basic healthcare coverage to 68.7% of residents. The city has 68 healthcare centers with defined territories and populations, organized into six Health Districts under the Municipal Health Secretariat. Campinas has 108 vaccination rooms: 70 are in public healthcare units, 23 in private services, and 15 in general hospitals and maternity wards offering vaccination services.

### 2.2. Sampling Strategy

This research identified households through a stratified and clustered sample by census sector, based on three stages: (a) the stratification of census sectors, (b) the formation of clusters of sectors based on the estimated number of live births, and (c) the systematic drawing and location of children in the cohorts of interest. The sample size per survey was calculated based on the following assumptions: hypothetical population size of 1,000,000 live births (LB); estimated prevalence of vaccination coverage of 70%, considering an estimation error of 5%; z = 1.96 for a 95% confidence interval; design effect by using clusters of census tracts, established at 1.4, obtaining the n of 452 children in each of the four socioeconomic strata, totaling four surveys (n = 1808 interviews).

Disproportionate allocation strategies were used to calculate sample weights for each stratum, avoiding biased estimates of the parameters of interest. Detailed sampling strategies and weight composition can be found in Barata et al., 2023 [6,11]. During household interviews, data on socioeconomic characteristics of families (household conditions, assets, presence of domestic workers, and household income) were collected, which resulted in a single socioeconomic indicator stratified into four groups: A (high), B (medium), C (lower-middle), and D (low), according to the classification proposed by the Brazilian Association of Research Companies (ABEP) [12].

This research gathered information on the date of administration of each vaccine according to the recommendations of the National Immunization Calendar in the first and second years of life, regardless of the timing and intervals between doses, considering application in the recommended period. Vaccines for the same disease and those administered in public and private sectors for children up to 24 months of age were included. Vaccination cards were photographed for subsequent analysis [11].

Reasons for non-vaccination were documented for each participating child and classified into three categories: (I) decision not to vaccinate (e.g., fear of adverse events/injections, lack of guidance from healthcare professionals, misinformation, “disease no longer exists”, disbelief in vaccines, COVID-19 pandemic, and other reasons); (II) difficulty accessing vaccination services (e.g., inconvenient operating hours, service location, lack of resources, inability to take time off work, family health issues, and other reasons); and (III) missed vaccination opportunities at healthcare services (e.g., vaccine/stock shortage, recommendation against vaccination, missing documents, long queues, closed vaccination rooms, or token systems for vaccination).

The categories of reasons for non-vaccination are defined as follows: In the decision not to vaccinate group (I), respondents who, at some point, chose not to administer one or more vaccines recommended by the Ministry of Health during the child’s first 24 months of life are included. In the difficulty accessing the vaccination service group (II), respondents who faced challenges in taking the child to the vaccination center belong to this group. In the missed opportunity group (III), respondents who, at some point, were unable to vaccinate their children despite visiting the vaccination center and encountering failures in vaccination services that became barriers to immunization are included.

Vaccination coverage estimates included all recorded doses on vaccination cards, regardless of timing and intervals between doses during the first 24 months of life [6,11]. Vaccination data were analyzed retrospectively from birth according to the schedules recommended by the PNI [12], yielding vaccination coverage percentages for each vaccine of the set of recommended doses and respective 95% confidence intervals, alongside the reported reasons for non-vaccination.

The proportions of vaccine hesitancy were calculated using Pearson’s chi-square test, considering *p* ≤ 0.05, comparing the four socioeconomic strata. The analysis was performed with a sample of respondents regarding the reasons for non-vaccination. A frequency analysis was conducted using a pre-weighted dataset, based on sample calibration variables.

Data analysis was conducted using STATA^®^ software version 14, with adjustments for sample calibration and survey weighting variables, employing the survey data analysis module [6]. The INCV was approved by the Research Ethics Committee of the Irmandade da Santa Casa de Misericórdia de São Paulo under co-substantiated opinion no. 4,380,019.

## 3. Results

In Campinas, this research interviewed 1775 caregivers of children born in 2017 and 2018, representing 98.2% of the planned interviews. Considering all doses recommended by the PNI, complete vaccination coverage was 63.1%. The coverage of valid doses was 43.3% and timely doses was 10.5%, which means that they were administered strictly within the intervals and periods of the vaccination schedule [6,13].

Table 1 presents the percentage distribution of the socioeconomic strata of respondents based on reasons for non-vaccination. In total, 50.2% missed vaccination opportunities in services occurred in stratum D, and 58.9% of families that reported difficulties accessing vaccination services belonged to strata C and D. In the group of reasons for fear and refusal due to lack of confidence in vaccines, the highest percentage was also concentrated in strata C and D, with lower socioeconomic status.

Table 2 shows the percentages of non-complete vaccination (%NV) among different strata, classified by reason groups, in addition to showing the proportion of those caregivers who did not vaccinate in each of the three categories and in each of the socioeconomic strata. Strata A and B had the highest %NV.

The highest percentages of non-complete vaccination were related to missed vaccination opportunities, at 16.4% (95% CI 12.4–21.4; N = 340) (group III). Despite the caregiver taking the child to the service for vaccination, the child was not vaccinated (Table 2). Moreover, 7.9% (95% CI 4.9–12.4; N = 87) of the families interviewed reported not vaccinating the child due to economic or access difficulties, and 1.7% (95% CI 0.9–2.9; N = 38) mentioned that they decided, at some point, not to administer one or more vaccines due to fear or distrust. Table 3, Table 4 and Table 5 show the reasons given by caregivers for not vaccinating their children (one or more vaccines), which are categorized into three groups. Since respondents were allowed to select more than one reason for not vaccinating, the same person could choose multiple reasons, which could refer to one vaccine or several.

In group I, fear (44.6%) was the most common reason reported, including fear of adverse events, injections, or the belief that vaccines could cause harm. Additionally, motivations related to the low-risk perception of diseases (26.2%) resulted in 71.1% of children in this category having incomplete vaccination. This reasoning is also linked to misinformation (19.0%) through fake news on social media, relatives, and friends, which is associated with 47% of vaccination failures (Table 3).

Advice from doctors not to vaccinate (23.7%) and contraindications during vaccine administration (27.3%) also drew attention, leading to 65.3% and 32.8% of children for each reason, respectively, not completing their vaccination schedules (Table 3).

The vaccines with the highest refusal/non-vaccination rates among the respondents in group I were Rotavirus (33.7%), Hepatitis B (31.1%), Yellow Fever (29.0%), BCG (23.5%), second dose of MMR (20.2%), and Meningococcal C (14.3%), as well as boosters for Pneumococcal (15.0%), Oral Polio (14.7%), DTP (14.6%), and Meningococcal C (14.3%).

The proportions of vaccines not received by the children in this group (Group I: refusal) suggest that the doses of the rotavirus and hepatitis B vaccines played a significant role in increasing the number of children with incomplete vaccination schedules in Group I.

Table 4 presents the reasons related to difficulties in accessing health services, many of which are associated with the social vulnerability of families (group II), such as restricted operating hours of vaccination rooms/units (63.4%), service location far from home, lack of money for transportation (48.3%), distance from family residences, and difficulties in taking time off work (5.3%) (Table 4). Physical and administrative barriers, along with misinformation about access, limited complete vaccination (%NV) by 14.2% and 70.6%, respectively.

It is observed that, despite the difficulties reported by caregivers in accessing healthcare services, families still seek alternatives to return to the service and remain persistent in vaccinating their children. Therefore, some end up not vaccinating the children at all, while others only delay the administration of the vaccines.

Of the caregivers who had ever decided not to vaccinate, a lower proportion of children had complete vaccination coverage in the first year of life (62.3% CI95% 32.0–85.3), while a higher percentage was observed in the second year of life (71.0% CI95% 53.8–83.7). There was no statistically significant difference between those who decided to vaccinate their children and those who did not.

For vaccines recommended for children over one year old, there was a difference in complete vaccination coverage between those who had difficulty taking the child to the vaccination center (44.5% CI95% 22.5–68.9) and those who did not (70.5% CI95% 62.3–77.6%) (*p* = 0.02).

Regarding missed opportunities, vaccination coverage among respondents was higher in the first twelve months of life (80.1% CI95% 69.1–87.9), with lower proportions of complete coverage in the second year of life (65.2% CI95% 55.7–73.6). There was no statistically significant difference in vaccination coverage between those who reported difficulties in accessing vaccination and those who did not.

In group II, vaccines with the highest non-vaccination rates included Rotavirus (24.9%), DTP boosters (29.8%), Pneumococcal (29.1%), Oral Polio/VOP (24.5%), Varicella (18.9%), and Meningococcal C (10%).

For caregivers of children who mentioned reasons related to missed vaccination opportunities (group III), the main reason was the lack of vaccines and/or materials for administration (80.0%), followed by having issues with access within the healthcare unit (24.4%), such as lack of personnel for administration, closed vaccination rooms, “not the day for specific vaccination”, restricted vaccination room hours, and construction works and renovations at vaccination sites (Table 5).

Although caregivers reported missed vaccination opportunities, many still make efforts to return for vaccination. As a result, some children miss their vaccinations entirely, while others experience delays in receiving them.

Although a lack of vaccines or materials was the primary reason reported for not vaccinating the child, 58.5% of the children who could not be vaccinated due to a lack of documentation did not complete their vaccination schedule. For those who mentioned a lack of vaccines or materials, 38.6% did not complete the vaccination schedule, which suggests that some caregivers may have returned to the service later. Reasons such as “lack of tokens” and “long vaccination queues” were barriers imposed by the management of healthcare units, contributing to the non-vaccination of 27.4% of children who attended the service.

The vaccines with the highest missed vaccination opportunities in services were as follows: the Meningococcal C booster (16.3%), DTP (16.2%), Pneumococcal (15.6%), Yellow Fever (15.4%), the second dose of MMR (14.6%), Hepatitis B (13.6%), Rotavirus (12.9%), BCG (12.7%), Varicella (12.4%), and the Oral Polio/VOP booster (12.1%), all showing rates above 10% for non-vaccination in this group.

## 4. Discussion

In Campinas, the complete vaccination coverage for the twelve vaccines recommended by the Ministry of Health’s vaccination schedule for the first 24 months of life was only 63.1%, falling short of the goals set by the National Immunization Program (PNI)—90% coverage for BCG and 95% for other vaccines. The greatest losses were observed among children over 12 months of age, despite the presence of a primary and hospital care network, a specific vaccination monitoring sector within the Municipal Health Department, and a structured Health Surveillance Department in the city.

When vaccination coverage is assessed based on the prescribed timing and intervals for each dose, these proportions are even lower, indicating greater vulnerability to these diseases. Vaccination coverage is a strategic indicator of the PNI as it makes it possible to assess herd immunity and reveal the level of protection in the population. The decline in vaccination rates highlights the emergence of unprotected groups, where viruses and bacteria may circulate, particularly affecting the most vulnerable individuals [10].

Vaccination coverage showed no statistically significant differences across socioeconomic strata, although greater access difficulties and missed vaccination opportunities were reported in higher proportions among the poorer population. Boing et al. (2024) emphasized racial inequalities and social vulnerabilities that interfere with vaccination coverage, demonstrating disparities in overcoming barriers to vaccination [10].

Incomplete vaccination schedules were mainly due to doses scheduled for the second year of the child’s life, such as booster doses for pneumococcal, meningococcal C, DTP, polio, and the second dose of MMR. As these doses were scheduled during the first year of the COVID-19 pandemic, some children born in 2018 were due to receive booster doses in 2020. Studies point to the impact of the COVID-19 pandemic in reducing routine vaccination coverage in Brazil and other parts of the world [14,15,16]. Other authors have reported lower coverage of booster doses due to reduced primary care use after the first year of life (such as child health checkups) and the lack of active outreach by health services to follow up with missing users, as they are not included in the vaccination coverage calculations of health units [14].

Vaccine hesitancy refers to the delay in accepting or refusing vaccination despite the availability of vaccination services. It is complex and context-specific, varying by time, location, and vaccine. It is influenced by factors such as complacency, convenience, and confidence [8,9]. Vaccine refusal involves a complexity and diversity of factors, from trust in vaccines, healthcare professionals, and decision-makers who approve vaccines to issues affecting access to immunobiological, such as the quality of immunization services and the individuals’ social context [9,17].

The survey provided proportions of vaccination in the cohort studied, both general and by socioeconomic stratum, as well as the context of hesitancy and the reasons reported by families.

After 2016, vaccination coverage for all vaccines offered by the PNI declined in all regions of Brazil, with a reduction in doses administered in 2019 [18]. The reemergence of vaccine-preventable diseases is imminent in the context of declining vaccination coverage and an increasing number of susceptible individuals, potentially causing new epidemics and posing challenges for health professionals inexperienced with these diseases. The success of immunization programs has led to the control, eradication, or elimination of such diseases [19,20,21].

The decision not to vaccinate (refusal) was mostly motivated by fear, linked to false information about severe adverse events and a lack of trust in vaccines. The engagement with false news and content on social media facilitates the spread of unchecked information, generating insecurity about vaccine effects and contributing to vaccine hesitancy [22]. A study in São Paulo indicated that parental anxiety and insecurity about potential adverse events significantly influence the decision not to vaccinate children [23].

A shift in vaccine perception has been observed globally, particularly in European countries. Studies reveal that negative perceptions of vaccine safety in France were based on multiple controversial aspects of immunobiological, including severe adverse events, the influence of medical professionals questioning vaccine efficacy, and hesitation among healthcare professionals who suggested that certain vaccines were unnecessary or that the national vaccination program had problems [24].

In many regions worldwide, higher levels of education are associated with a lower likelihood of reporting positive sentiments about vaccines, related to trust [24]. In Brazil, the rapid flow of uncontrolled information has reinforced the anti-vaccine movement, previously not very expressive in the country, particularly among the poorer population groups.

In the U.S., declining vaccination coverage has been associated with recent outbreaks of measles, mumps, and whooping cough [21], a non-negligible risk in Brazil [5]. This underscores the challenge of health communication in vaccination surveillance actions and control strategies [22,23].

The perception of no risk from vaccine-preventable diseases in the country and anti-vaccine movements in developed countries have particularly influenced the wealthier classes in Brazil over the last decade. However, the dissemination of false news during the COVID-19 pandemic expanded this perception to poorer populations, reinforced by the Ministry of Health regarding mistrust in vaccination.

In addition to decisions not to vaccinate and a lack of perceived disease risk, access difficulties stand out, particularly associated with the lack of financial resources for transportation and the inability to leave work to visit health units. These barriers, linked to social vulnerability, contributed to incomplete vaccination schedules. Socioeconomic strata C and D, which predominantly rely on public vaccination services, reported these challenges more frequently. Social inequalities impacting vaccination are based on disparities in wealth, education, and geographic access, forming significant barriers to vaccination. Poor populations, residents of underprivileged areas, and Black and mixed-race individuals show lower percentages of complete immunization, which increases the risk of vaccine-preventable infections [7,12,25].

Factors that reinforce vaccine hesitancy, such as fear and risk perception, highlight the need for more effective health communication strategies. Educational campaigns and counseling by healthcare professionals can help reduce misinformation and increase confidence in vaccines. Additionally, ensuring that healthcare professionals are better trained to address caregivers’ concerns and clarify doubts can help mitigate these barriers.

Respondents most frequently reported problems related to infrastructure, human resources, vaccine and supply shortages, and management challenges in basic health units, resulting in missed vaccination opportunities, as indicated by similar studies [10,11]. These administrative and management barriers and structural issues in vaccination rooms and health team training partially reflect the underfunding of the Unified Health System (SUS). Additionally, they highlight challenges in ensuring access to healthcare services, particularly vaccination, alongside structural issues in vaccination rooms and health teams. Addressing these issues requires strengthening primary care, expanding and training health teams to enhance their knowledge of the national vaccination schedule (MS), improving work processes, and upgrading the infrastructure of vaccination rooms.

Infrastructure-related challenges can create significant barriers to immunization. For example, caregivers who work long hours may struggle to find time to take their children for vaccination, while those facing financial constraints may be unable to afford transportation. Additionally, if vaccination sites are under renovation or closed on certain days, families may miss opportunities to immunize their children. A lack of clear information about vaccine schedules and eligibility can further contribute to delays or missed vaccinations.

Missed vaccination opportunities stem from service management issues, a lack of welcoming environments, and inflexible teams in offering vaccinations at feasible times and schedules. Rigid structures in some units, such as limited token distribution and the absence of family medical records, hinder vaccination. Revisiting routines to prioritize vaccination opportunities, reorganizing queues, and conducting home visits to identify absentees are necessary strategies.

The unavailability of vaccines and supplies, the lack of trained professionals, and restrictions on vaccination center hours cause delays in immunization and may compromise coverage targets, increasing the risk of outbreaks. Also, long waiting times, limited daily doses, and bureaucratic requirements hinder access, particularly affecting vulnerable populations. Inadequate guidance from healthcare professionals can reinforce vaccine hesitancy, further reducing vaccination rates. These structural and administrative barriers reinforce inequalities and require actions to improve the accessibility and efficiency of vaccination services.

Addressing these management challenges reaffirms the SUS principles of universality, equity, and comprehensiveness. Flexible vaccination room hours and the strategic location of health units directly impact child vaccination rates. Family health teams and community health agents play a critical role in identifying vulnerable families and supporting strategies to restore vaccination coverage.

The COVID-19 pandemic added significant challenges to maintaining vaccination coverage, as routine care was suspended to meet the demand for symptomatic respiratory cases, impacting childhood vaccination. Reductions in measles vaccination rates among children under one year of age in Brazil were observed during this period. These declines were more prominent in populous and unequal municipalities in the North and Northeast regions, with lower Primary Health Care coverage, revealing severe health inequities when access to health services is affected [5,15].

Vaccination coverage surveys can provide precise information on territorial coverage, homogeneity, reasons for non-vaccination, and strategic PNI indicators for controlling and monitoring of vaccine-preventable diseases in the country. The monitoring of childhood vaccination coverage and investigations into reasons for non-vaccination should be ongoing. Although this monitoring is already carried out through the SUS information system, it needs improvements, including improving the structure of health units, strengthening the training of health teams, and increasing funding to ensure better access to vaccination services.

Among the limitations of the study are the difficulties in accurately listing the main reasons for non-vaccination in a single interview, due to the complexity of the phenomenon of vaccine hesitancy, often associated with socioeconomic difficulties and missed opportunities in services. In a survey, there are possible selection biases in the case of closed houses, families not found, or those who refused to be interviewed. However, these losses were small (6.0%) and we do not believe that they compromised the external validity of the sample. The reasons for non-vaccination often overlap. On the other hand, photographic records of vaccination cards for later analysis provide greater precision of the coverage data for each vaccine in the calendar.

## 5. Conclusions

Vaccination coverage among children up to 24 months of age in Campinas was similar across socioeconomic strata, although access barriers and missed vaccination opportunities were more significant among the poorest groups.

This population-based vaccination survey showed the points that must be addressed to expand the complete coverage of vaccination for the population in the first 24 months of life. Missed vaccination opportunities in health services were the most frequent, while the fear of adverse events and injections, a lack of trust in vaccines, a lack of perceived risk of diseases, and the influence of false information played an important role in low vaccination coverage. Economic and social difficulties also further contributed to challenges in accessing vaccination services. Population surveys can provide relevant information about the context of vaccination difficulties and the reasons for drops in coverage that can help reformulate health policies, creating strategies to intervene in the barriers that tend to influence refusals or delays in vaccinations.

## Figures and Tables

**Table 1:** Percentage distribution of socioeconomic strata of respondents regarding reasons for non-vaccination of children born in 2017 and 2018, Campinas, SP.

Stratum	Group I Refusal/Fear (%)	Group II Difficulty/Access (%)	Group III Missed Opportunity (%)
A	12.7	26.5	5.3
B	17.8	14.7	20.7
C	43.7	29.9	23.8
D	25.8	28.9	50.2
Total	100% (38)	100% (87)	100% (340)

**Table 2:** Proportion of caregivers reported not having administered one or more vaccines and percentage of non-complete vaccination (%NV) according to socioeconomic strata within groups of reasons in children born in 2017 and 2018, Campinas, SP.

	Group I		Group II		Group III		Total
	Refusal/Disinformation		Access/Difficulty		Missed Vaccination Opportunity		
Stratum *	%	NV (%)	%	NV (%)	%	NV (%)	NV (%)
	CI95%	CI95%	CI95%	CI95%	CI95%	CI95%	CI95%
**A**	0.8	76.6	8.1	93.7	3.3	41.5	44.6
	(0.3–2.1)	(40.0–94.1)	(2.2–25.9)	(65.4–99.1)	(1.6–7.0)	(26.2–58.6)	(19.6–72.6)
**B**	1.9	57.6	7.2	45.3	21.1	37.3	35.2
	(0.7–4.6)	(33.0–79.0)	(3.2–15.7)	(12.2–83.1)	(16.8–26.3)	(23.2–54.1)	(23.6–48.9)
**C**	2.9	58.7	9.3	56.3	15.4	33.3	37.5
	(0.9–8.4)	(16.5–91.1)	(3.7–21.4)	(19.0–87.6)	(8.7–25.6)	(20.4–49.3)	(29.4–46.4)
**D**	1.3	24.0	7.0	26.6	25.3	38.3	31.3
	(0.6–3.0)	(6.9–57.2)	(4.3–11.2)	(10.0–54.2)	(19.3–32.4)	(24.8–53.9)	(23.8–39.9)
**Total**	1.7	51.8	7.9	56.0	16.4	37.1	37.0
	(0.9–2.9)	(28.2–74.7)	(4.9–12.4)	(31.6–77.8)	(12.4–21.4)	(28.8–46.3)	(28.9–45.8)

* Stratum A—less vulnerable, D—more socially vulnerable.

**Table 3:** Detailed reasons for the decision driven by fear and refusal (Group I) of caregivers for not administering one or more vaccines and the percentage of non-vaccination (%NV) in children up to 24 months of age in Campinas, SP.

	N (%)	(CI95%)	%NV
Incomplete coverage	19 (51.8)	(28.2–74.7)	
**Reasons**			
Fear (side effects, injection)	16 (44.6)	(18.9–73.5)	71.1
“Doctor advised not to vaccinate”	11 (23.7)	(11.2–43.4)	65.3
Disinformation ^1^	9 (19.0)	(7.6–40.1)	47.0
Contraindication	8 (27.3)	(11.0–53.4)	32.8
Risk perception ^2^	7 (26.2)	(13.5–44.6)	71.1
Pandemic	2 (4.6)	(0.9–19.4)	0
Others ^3^	4 (18.9)	(6.9–42.0)	72.1

^1^ Disease no longer exists, fake news, advice from a relative or friend, considers the vaccine ineffective and outdated. ^2^ Does not believe in the vaccine, sees no need. ^3^ Others: Does not know, did not administer flu vaccines, multiple reasons, sick family.

**Table 4:** Caregivers’ difficulty accessing health services (Group II) and not administering one or more vaccines, and the percentage of non-vaccination (%NV) in children up to 24 months of age in Campinas, SP.

	N (%)	(CI95%)	%NV
Incomplete coverage	29 (56.0)	(31.1–77.6)	
**Reasons**			
Inconvenient hours	39 (63.4)	(40.0–81.8)	65.5
Distant clinic, lack of money	45 (48.3)	(23.3–74.2)	58.4
Sick child	11 (12.0)	(3.2–36.2)	15.8
Physical and administrative barriers ^1^	12 (11.5)	(4.1–28.2)	14.2
Lack of information about access ^2^	9 (6.4)	(2.2–16.7)	70.6
Employer does not allow time off work	7 (5.3)	(2.1–12.7)	31.5
Pandemic	5 (2.3)	(0.8–6.8)	78.7
Others ^3^	7 (2.6)	(0.7–9.7)	24.8

^1^ Change of health clinic, no vaccination booklet/medical record, clinic under renovation. ^2^ Does not know when to go, claims lack of information from the local government. ^3^ Others: Sick relative, does not know.

**Table 5:** Missed opportunity for vaccination in healthcare services (Group III) and not administering one or more vaccines, and the percentage of non-complete vaccination (%NV) in children up to 24 months of age in Campinas, SP.

	N (%)	(CI95%)	%NV
Incomplete coverage	98 (37.1)	(28.8–46.3)	
**Reasons**			
Missed vaccines and supplies	270 (80.0)	(73.1–85.5)	38.6
Team/structure/management issues ^1^	92 (24.4)	(18.0–32.2)	24.9
Health professional’s recommendation	27 (6.8)	(3.5–13.0)	26.7
“No more vaccination passwords”/queues	35 (8.6)	(5.1–14.1)	27.4
No documentation ^2^	13 (4.3)	(2.0–9.1)	58.5
Pandemic	4 (0.6)	(0.2–1.7)	12.0
Others ^3^	5 (3.8)	(1.3–10.6)	38.4

^1^ No professional available, vaccination room closed, “not the vaccination day”. Vaccination room hours, construction, and renovation. ^2^ No medical record at the service, no vaccination booklet. ^3^ Others: Does not know, multiple reasons. Note: More than one reason was mentioned during the interview.

## Data Availability

All authors have shared and agree to the statements contained in the MDPI Research and Publication Ethics. Data and results of the Survey are public and available in the "Relatório de Inquerito National de Cobertura Vacinal Brasil, 2024 available at: https://bvsms.saude.gov.br/bvs/publicacoes/inquerito_cobertura_vacinal_urbanas.pdf (accessed on 22 January 2025).

## Abbreviations

The following abbreviations are used in this manuscript:

MDPI	Multidisciplinary Digital Publishing Institute
DOAJ	Directory of open access journals
PNI	National Immunization Program
SUS	Unified Health System
INCV	National Vaccination Coverage Survey
HDI	Human Development Index
ESF	Family Health Strategy
PPT	Proportional allocation procedures
ABEP	Brazilian Association of Research Companies
DTP	Diphtheria-tetanus-pertussis
MMR	Measles-Mumps-Rubella
BCG	Bacilo Calmette Guérin
NV	Non-vaccination
